# Lung cancer-associated fibroblasts-mediated collagen deposition drives mediastinal lymph node metastasis in non-small cell lung cancer

**DOI:** 10.3389/fonc.2025.1597585

**Published:** 2025-06-12

**Authors:** Caoyang Chen, Yuqian Feng, Frankie Chi Fat Ko, Sze Kwan Lam, Sheng Yan, James Chung Man Ho

**Affiliations:** ^1^ Department of Cardiothoracic Surgery, Wuhan Fourth Hospital, Wuhan, Hubei, China; ^2^ Division of Respiratory Medicine, Department of Medicine, School of Clinical Medicine, The University of Hong Kong, Hongkong, Hong Kong SAR, China; ^3^ Department of Respiratory and Critical Care Medicine, Union Hospital, Tongji Medical College, Huazhong University of Science and Technology, Wuhan, Hubei, China

**Keywords:** cancer-associated fibroblasts, lymphangiogenesis, mediastinal lymph node metastasis, NSCLC, orthotopic xenograft model

## Abstract

**Introduction:**

Metastasis to mediastinal lymph nodes signifies an advanced stage of non-small cell lung cancer (NSCLC) and presents significant clinical challenges. Cancer-associated fibroblasts (CAFs) within the tumor microenvironment (TME) play a crucial role in tumor progression by promoting growth and invasion. However, the specific contributions of lung CAFs to mediastinal lymph node metastasis in NSCLC remain poorly understood. Moreover, no therapeutics currently target CAFs to combat mediastinal lymph node metastasis in NSCLC. This study aims to elucidate the precise roles of CAFs in these complex processes and to investigate innovative therapeutic strategies that target CAFs to suppress metastasis to mediastinal lymph nodes.

**Methods:**

Normal human lung fibroblasts (MRC-5) were directly co-cultured with NSCLC cell lines (H358 and HCC827) to generate lung CAFs. These activated CAFs were identified using immunofluorescence, flow cytometry, and Western blotting. To model human mediastinal lymph node metastasis, orthotopic xenograft (OX) models were established by intrathoracically injecting NSCLC cells into the left lung of mice, either alone or in combination with lung CAFs. The viability of cancer cells and lung CAFs post-treatment with ABT-199, a Bcl-2 inhibitor, was evaluated using MTT assays. The therapeutic efficacy of ABT-199 was further assessed through oral gavage in OX models, focusing on its potential to prevent metastasis to mediastinal lymph nodes. Tumor growth was monitored longitudinally using micro-computed tomography (CT), and treatment response was evaluated in accordance with RECIST 1.1 criteria. Primary tumors and mediastinal lymph node metastases were analyzed using hematoxylin and eosin (H&E) staining for general morphology, supplemented by immunohistochemistry/immunofluorescence to detect specific protein markers. Fibrillar collagen deposition within the TME was quantified using picrosirius red staining (PRS).

**Results:**

Activation of α-SMA, accompanied by a significant increase in Col 1A1 expression in MRC-5 cells, was successfully induced through a direct 14-day co-culture with NSCLC cell lines. Histological analysis revealed increased fibrotic tissue formation, enhanced fibrillar collagen deposition, peritumoral lymphangiogenesis, and mediastinal lymph node metastasis in CAFs-enriched orthotopic xenograft (OX) tumors compared to CAFs-devoid OX tumors. The viability of lung CAFs was dose-dependently inhibited by ABT-199 *in vitro*. A two-week treatment with ABT-199 (100mg/kg) led to a significant reduction in lung CAFs and lymphangiogenesis in the CAFs-enriched OX model. Furthermore, an eight-week treatment with ABT-199 (100mg/kg) significantly reduced fibrillar collagen deposition and inhibited the number of metastases to mediastinal lymph nodes in both CAFs-devoid and CAFs-enriched OX models.

**Conclusions:**

In NSCLC, cancer cells induce the differentiation of resident normal lung fibroblasts into lung CAFs. These CAFs secrete excessive collagens within the TME, thereby promoting tumor lymphangiogenesis and facilitating metastasis to mediastinal lymph nodes. Our findings, based on modified OX models, suggest that targeting lung CAFs could effectively attenuate lymphatic dissemination in NSCLC.

## Introduction

Metastasis to mediastinal lymph nodes marks a pivotal deterioration point in non-small cell lung cancer (NSCLC) progression, substantially worsening patient prognosis and complicating treatment strategies (1, 2). While early research focused predominantly on cancer cell-autonomous mechanisms of metastasis, contemporary understanding recognizes the tumor microenvironment (TME) as an active participant in metastatic dissemination (3, 4). Among TME components, cancer-associated fibroblasts (CAFs) have emerged as critical mediators of metastatic progression through their multifaceted roles in extracellular matrix remodeling, growth factor secretion, and immune modulation (5–7).

Recent single-cell transcriptomic analyses have revealed the abundant presence of CAFs not only in primary NSCLC tumors (8) but also within lymph node metastases (9). Clinical studies of over 1,000 NSCLC cases have established a strong correlation between CAFs abundance and poor clinical outcomes, including increased metastatic frequency and reduced survival (10). Functional evidence from xenograft models demonstrates that CAFs can potentiate tumor growth and metastatic spread, as shown in both subcutaneous NSCLC models (11) and orthotopic esophageal cancer systems (12). However, the specific mechanisms by which lung CAFs facilitate mediastinal lymph node metastasis remain poorly understood, hampered by the lack of physiologically relevant experimental models (13).

Orthotopic xenograft models in the lung, rather than subcutaneous sites, offer a more natural TME for cancer cell growth (14–17) and better replicate mediastinal lymph node metastasis in NSCLC (18, 19). However, the commonly used murine lung cancer models have lower CAFs content compared to human counterparts (13). Incorporating additional CAFs into these models enhances their fidelity to the human TME (13), underscoring the need for CAFs supplementation in orthotopic xenografts.

Human lung CAFs, isolated based on their morphological characteristics and lack of lineage-specific markers (e.g., EpCAM, CD31, CD45) (20), vary in subtype and function (10, 21). Previous studies using co-injection methods with human CAFs from different tumor types have illuminated CAFs biological roles but face challenges in reproducibility (22). Moreover, the heterogeneity of human CAFs (5, 23, 24), complicates understanding their specific functions in mediastinal lymph node metastasis and limits preclinical trials.

To address these challenges, we developed an optimized orthotopic xenograft system incorporating activated lung CAFs generated through controlled co-culture conditions. This model offers several advantages:

1. Preserves the natural lung TME context for tumor-stroma interactions.
2. Maintains consistent CAFs populations through defined activation protocols.
3. Recapitulates key features of human NSCLC metastasis.
4. Provides a platform for evaluating CAFs-targeted therapies.

Our approach bridges a critical gap in NSCLC metastasis research by establishing a reliable, clinically relevant model system that enables mechanistic investigation of CAFs-mediated lymph node metastasis and preclinical evaluation of stroma-directed therapeutic strategies. This model holds particular promise for developing interventions targeting the CAFs-dependent steps of metastatic progression, an area currently lacking effective clinical approaches.

## Materials and methods

### Cell lines

Human NSCLC cell lines (H358, HCC827, H1993) and human normal fibroblasts (MRC-5) were used in this study and purchased from the American Type Culture Collection (ATCC, Manassas, VA, USA). H358, HCC827, H1993 and MRC-5 cells were cultured in RPMI-1640 medium Gibco^®^ (Thermo Fisher Scientific, Inc., Waltham, MA, USA) supplemented with 10% fetal bovine serum (FBS; Gibco^®^; Thermo Fisher Scientific, Inc.) in a humidified atmosphere of 5% CO2 at 37°C. All cell cultures underwent regular testing for *Mycoplasma* contamination and consistently yielded negative results.

### Generation of lung CAFs by direct co-culture

Before co-culture, over 80% cell viability was confirmed by Trypan blue staining. H358, HCC827 or H1993 and MRC-5 cells were counted and seeded at a ratio of 1:3-1:4 for direct co-culture in 150 cm^2^ flasks in RPMI-1640 medium Gibco^®^ supplemented with 10% FBS in a humidified atmosphere of 5% CO2 at 37°C (25). The medium was changed every other day. Trypsin was used to adjust the percentage of co-cultured cells. After adding trypsin and incubation at 37°C for about 1 minute, most MRC-5 cells became non-adherent and were washed with PBS for the next step of co-culture.

### Identification of lung CAFs by immunofluorescence

After 14-day direct co-culture, cells were seeded on sterilized cover slips in 12-well plates in a humidified atmosphere of 5% CO2 at 37°C. Upon reaching 70-80% confluence in a single layer, cells were fixed by 4% paraformaldehyde and permeabilized by 0.5% Triton X-100 in PBS (PBST) (25). Cells were blocked in blocking buffer (3% bovine serum albumin in PBST) at room temperature for 30 minutes. Primary antibodies (α-SMA, 1A4, ab7817, abcam, 1:200; α-SMA, D4K9N, #19245, Cell Signaling Technology, Inc., 1:200; CK7, D1E4, #4465, Cell Signaling Technology, Inc., 1:500; VEGF-C, E-6, sc-374628, Santa Cruz, 1:200; VEGF-D, C-12, sc-373866, Santa Cruz, 1:50; PDGFRβ, D-6, sc-374573, Santa Cruz, 1:200; PDPN, 18H5,sc-59347,Santa Cruz, 1:200); were diluted to in blocking buffer and incubated at 4°C overnight. Alexa Fluro™ 568 donkey anti-mouse IgG (H+L) (#A-10037, Invitrogen; Thermo Fisher Scientific, Inc., 1:500) and Alexa Fluro™ 488 goat anti-rabbit IgG (#A-10034, Invitrogen; Thermo Fisher Scientific, Inc., 1:500) were diluted in blocking buffer and incubated at 4°C in the dark for 1 hour. Prolong^®^ Gold anti-fade reagent (Invitrogen; Thermo Fisher Scientific, Inc.) containing 4’,6-diamidino-2-phenylindole (DAPI) was added to the cover slips. Slides were sealed with nail polish. Images were captured by a fluorescence microscope (Nikon, Tokyo, Japan).

### Identification of lung CAFs by western blot

Cells were collected and lysed in sodium dodecyl sulphate (SDS) lysis buffer (1% SDS, 10 mM HEPES, pH7.0, 2 mM MgCl2, universal nuclease 20 U/ml). Total cellular protein concentration was measured using a BCA assay kit (Thermo Fisher Scientific Inc.). Equal amounts of the protein samples were subjected to 10% SDS-PAGE and transferred to nitrocellulose blotting membranes (GE Healthcare). The membranes were then blotted with primary antibodies α-SMA, [1A4, ab7817, abcam, 1:200]; Col 1A1[E3E1X, #66948, Cell Signaling Technology, Inc., 1:1000]; GAPDH [sc-47724, Santa Cruz Biotechnology], 1:1000) at 4°C overnight, then incubated with the secondary antibody (horse anti-mouse IgG, HRP-linked antibody (#7076, Cell Signaling Technology, Inc.,1:1000); goat anti-rabbit IgG, HRP-linked antibody (#7074, Cell Signaling Technology, Inc.,1:1000)) for 1h at room temperature. Proteins were visualized using enhanced chemiluminescence exposed on autoradiograph film and developed using standard methods previously described (26).

### Quantification of lung CAFs by flow cytometry

After identification of α-SMA^+^ fibroblasts by immunofluorescence, co-cultured cells were harvested in ice cold phosphate buffered saline (PBS) and around 5×10^5^ cells allocated per tube. To stain cancer cells, PE anti-human CD326 (EpCAM) antibody (9C4, #324206, Biolegend, 1:200) and PE mouse IgG2b, Isotype Ctrl antibody (MPC-11, #400312, Biolegend, 1:500) were diluted in ice cold fluorescence-activated cell sorting (FACS) buffer and incubated on ice for 30 minutes in the dark. After washing with FACS buffer, cells were fixed and permeabilized by buffer (4% paraformaldehyde and 0.5% Triton X-100 in PBS) on ice for 30 minutes in the dark. To stain α-SMA^+^ fibroblasts, anti-α-SMA antibody (1A4, ab7817, abcam, 1:200) was diluted in washing buffer and incubated at 4°C for 1 hour in the dark. After washing with FACS buffer, goat anti-mouse IgG-FITC (sc-2010, Santa Cruz Biotechnology, 1:500) was diluted in washing buffer and incubated on ice for 30 minutes in the dark. Cells were then acquired on CytoFLEX (Beckman Coulter, Inc., USA) for flow cytometry analysis. Data analysis was performed using FlowJo™ (BD biosciences).

### Isolation of lung CAFs by FACS

Cell sorting was performed as described previously (25). EpCAM was utilized as a surface marker to isolate H358 or HCC827 cells from MRC-5 cells. After 14 days of co-culture, cells were incubated with PE anti-human CD326 (EpCAM) antibody (9C4, #324206, Biolegend, 1:200). PE mouse IgG2b, Isotype Ctrl antibody (MPC-11, #400312, Biolegen, 1:500) was added to cells as a control for gating during cell sorting. EpCAM^+^ and EpCAM^-^ cells were isolated by MoFlo XDP (Beckman Coulter). Data analysis was also performed on FlowJo™.

### Establishment of orthotopic xenograft mouse model by intrathoracic injection

Female BALB/cAnN-nu (nude) mice (4–6 weeks) were used for intrathoracic injections. After acclimatization for one day, mice were anesthetized by intraperitoneal administration of ketamine (100 mg/kg) and xylazine (10 mg/kg). H358 or HCC827 cells (2×10^6^) with or without lung CAFs (0.5×10^6^) were re-suspended with growth factor reduced Matrigel^®^ (Corning) in PBS (total volume < 40 μl) using insulin syringes (BD) and kept on ice. Subsequently, cells were directly injected into the left lung of each mouse (15). The technique of intrathoracic injection is described in greater detail below. After confirming sufficient anesthesia by toe pinch, the mouse was placed in a lateral decubitus position on a warm blanket. Skin was disinfected with 10% betadine solution, then wiped off using 70% alcohol cotton swabs. The left scapula was located by gentle determination of the subscapular angle. Then along the subscapular angle, the upper and lower ribs were felt with the ventral side of the left index finger. The point at which the perpendicular line of the subscapular angle intersected the horizontal line of the ribs was the injection site. The needle was advanced rapidly to a depth of approximately 5 mm into the left lobe of the lung between the 6th and 7th ribs, the plunger withdrawn to exclude puncture of any blood vessels and the cell suspension injected slowly. After complete injection, the needle was maintained in the lung for 15 seconds to avoid leakage of the injected material, then quickly removed and pressure applied to the area with cotton swabs soaked with 70% alcohol until hemostasias was achieved. The mouse was monitored closely until full recovery from anesthesia. All mice experiments were approved by the Committee on the Use of Live Animals in Teaching and Research, The University of Hong Kong (CULATR 5580-20).

### Evaluation of tumor formation by micro-computed tomography

Before performing micro-CT (Bruker, SkyScan1276) scan, mice were deeply anesthetized by intraperitoneal injection of ketamine (100 mg/kg) and xylazine (10 mg/kg). Mice were then placed prone in the micro-CT chamber and wrapped in a bed using paper adhesive tape to avoid any movement (27). The micro-CT scan time was adjusted to 45 seconds per mouse. After scanning, mice were placed in a warm incubator to recover. Micro-CT scan was performed weekly to monitor tumor formation and progression. Images were analyzed by Dataviewer (Bruker) and Fiji (ImageJ, NIH). Quantification of tumor size was based on the Response Evaluation Criteria in Solid Tumor (RECIST 1.1) (28).

### Collection of tumor and mediastinal lymph node metastasis

Mice were euthanized with intraperitoneal injection of pentobarbital. A lower abdominal incision was made to expose the organs and to detect metastasis. Mice were exsanguinated by cutting the abdominal aorta and inferior vena cava. Diaphragm was carefully removed to detect primary tumor, pleural effusions, and intrathoracic metastasis. Then rib cage with sternum was cut off to expose the heart, lungs, and mediastinum. 25G needle attached with a syringe containing 5 ml cold PBS was inserted into the right ventricle of the heart to perfuse the lung tissue. After removal of heart, the whole mediastinum with lungs were resected and washed in cold PBS. The samples were immediately fixed in 4% paraformaldehyde for 24–48 hours followed by embedding in paraffin (15).

### Confirmation of primary tumor and mediastinal lymph node metastasis by H&E and immunohistochemistry

Formalin-fixed paraffin embedded (FFPE) tissues were cut at 4-6 μm, dewaxed in xylene, and rehydrated through graded ethanol solutions. For hematoxylin & eosin (H&E) staining, slides were immersed into hematoxylin solution for 3–5 minutes and eosin solution for 1 minute. Primary tumor and mediastinal lymph node metastasis were examined under a microscope. For IHC staining, following deparaffinization and rehydration, slides were boiled in microwave for antigen retrieval (10 mM sodium citrate, 0.05% Tween 20, pH 6). Then, slides were immersed into blocking buffer (BLOXALL^®^ SP-6000-100, Vector Laboratories, Inc.) for at least 15 minutes to block endogenous peroxidase and alkaline phosphatase. After permeabilization (0.5% Triton X-100 in PBS) and non-specific binding blocking (2.5% normal horse serum, MP-7500-50, Vector Laboratories, Inc.), slides were stained with anti-CK7 (D1E4, #4465, Cell Signaling Technology Inc. 1:500; VEGF-C, E-6, sc-374628, Santa Cruz, 1:200; VEGF-D, C-12, sc-373866, Santa Cruz, 1:50);, anti-Ki-67 (8D5, #9449, Cell Signaling Technology Inc. 1:500), anti-α-SMA (D4K9N, #19245, Cell Signaling Technology Inc. 1:200) or anti-lyve-1 (NB100–725B, NOVUS biologicals, 1:200) antibodies, and then processed with the ImmPRESS™ HRP Universal Antibody (Horse Anti-Mouse/Rabbit IgG) Polymer Detection Kit (MP-7500-50, Vector Laboratories, Inc.) and Liquid DAB+ Substrate Chromogen System kit (K3468, DAKO). Slides were counterstained with hematoxylin and dehydrated through graded alcohols and xylene. Mounting media were added to seal slides with coverslips (29).Images were captured by microscope (Nikon, Tokyo, Japan). CK7/Ki-67 positive in lymph node was diagnosed as metastasis (30). Data were analyzed by Fiji (ImageJ, NIH).

### Quantification of α-SMA area index and lymphangiogenesis by IF/IHC

Following deparaffinization, rehydration, antigen retrieval, permeabilization (0.5% Triton X-100 in PBS) and non-specific binding blocking (2.5% normal horse serum, MP-7500-50, Vector Laboratories, Inc.; 2.5% normal goat serum, 50062z, Thermo Fisher; 2.5% bovine serum albumin solution, Sigma), slides were stained with anti-α-SMA (D4K9N, #19245, Cell Signaling Technology Inc.; 1A4, ab7817, abcam,1:200) and anti-lyve-1 (NB100–725B, NOVUS biologicals, 1:500) antibodies overnight at 4°C. Alexa Fluro™ 568 donkey anti-mouse IgG (H+L) (#A-10037, Invitrogen; Thermo Fisher Scientific, Inc.) and Alexa Fluro™ 488 goat anti-rabbit IgG (#A-10034, Invitrogen; Thermo Fisher Scientific, Inc.) were diluted in blocking buffer and incubated at 4°C in the dark for 1 hour. Prolong^®^ Gold anti-fade reagent (Invitrogen; Thermo Fisher Scientific, Inc.) containing DAPI was added to the cover slips. Slides were sealed with nail polish. Images were captured by a fluorescence microscope (Nikon, Tokyo, Japan). α-SMA scoring was evaluated as an “Area Index,” calculated by Fiji (ImageJ, NIH). To ensure that the whole tumor was evenly evaluated, at least three or more fields including stromal cells were carefully selected to evaluate CAFs. The mean value obtained from each sectioned tissue was defined as the α-SMA^+^ area index (31). The hot spots of lyve-1 positive lymph vessels were measured as lymphangiogenesis at 200x magnification (29).

### Quantification of fibrillar collagen deposition area index by picrosirius red staining

Fibrillar collagen deposition was conducted using Picrosirius red staining kit (#24901, Polysciences, Inc.) (32). Images were captured by microscope (Nikon, Tokyo, Japan). Collagen deposition (red) was evaluated as an “Area Index,” calculated by Fiji (ImageJ, NIH).

### 3-(4,5-dimethylthiazol-2-yl)-2,5-diphenyltetrazolium bromide (MTT) assay

Around 5000 cells were seeded in a 96-well plate and incubated in a humidified atmosphere of 5% CO2 at 37°C for 24 hours (26). ABT-199 was added at concentrations from 0-40 μM and incubated for 24 hours. 20 μl of 2.5 mg/ml MTT was added to each well and incubated at 37°C for 1 hour. Media were carefully removed and 100 μl DMSO added to each well. Absorbance was read at OD590 nm.

### ABT-199 treatment in mouse model

Tumor formation was confirmed by micro-CT 7 days after injection. Mice were and randomly assigned to two groups and treated with vehicle or ABT-199 (100 mg/kg) (33) daily by oral gavage for 2 weeks, 4 weeks or 8 weeks (33, 34). ABT-199 (Venetoclax) was purchased from MedChemExpress (MCE^®^ Cat. No.: HY-15531/CS-1155, Purity:99.96%). ABT-199 (10 mg/ml) was formulated in 5% DMSO, 40% PEG 300, 5% Tween 80, and 50% dH2O.

### Statistical analysis

Data collected from triplicate experiments are presented as Mean ± SEM. Comparison of different groups was performed using Student’s paired or unpaired two-tailed t-test, one-way ANOVA and two-way ANOVA by GraphPad Prism (v6.0). Statistical significance (n.s.: not significant, *: p <0.05, **: p <0.01, ***: p <0.001, ****: p <0.0001) indicates comparison with control.

## Results

### Human NSCLC cells induce the transformation of normal lung fibroblasts into α-SMA^+^ CAFs through direct co-culture

To investigate whether human lung fibroblasts can be transformed into CAFs by cancer cells, we co-cultured MRC-5 cells with H358, HCC827, and H1993 cells for 14 days. Following co-culture, MRC-5 cells exhibited significant morphological alterations, including prominent filament formation, as observed by light microscopy (**Supplementary Figure S1a**) and immunofluorescence (IF) microscopy (**Supplementary Figure S1b**), compared to monocultured MRC-5 controls (**Supplementary Figures S1a–c**).

**Figure 1:**
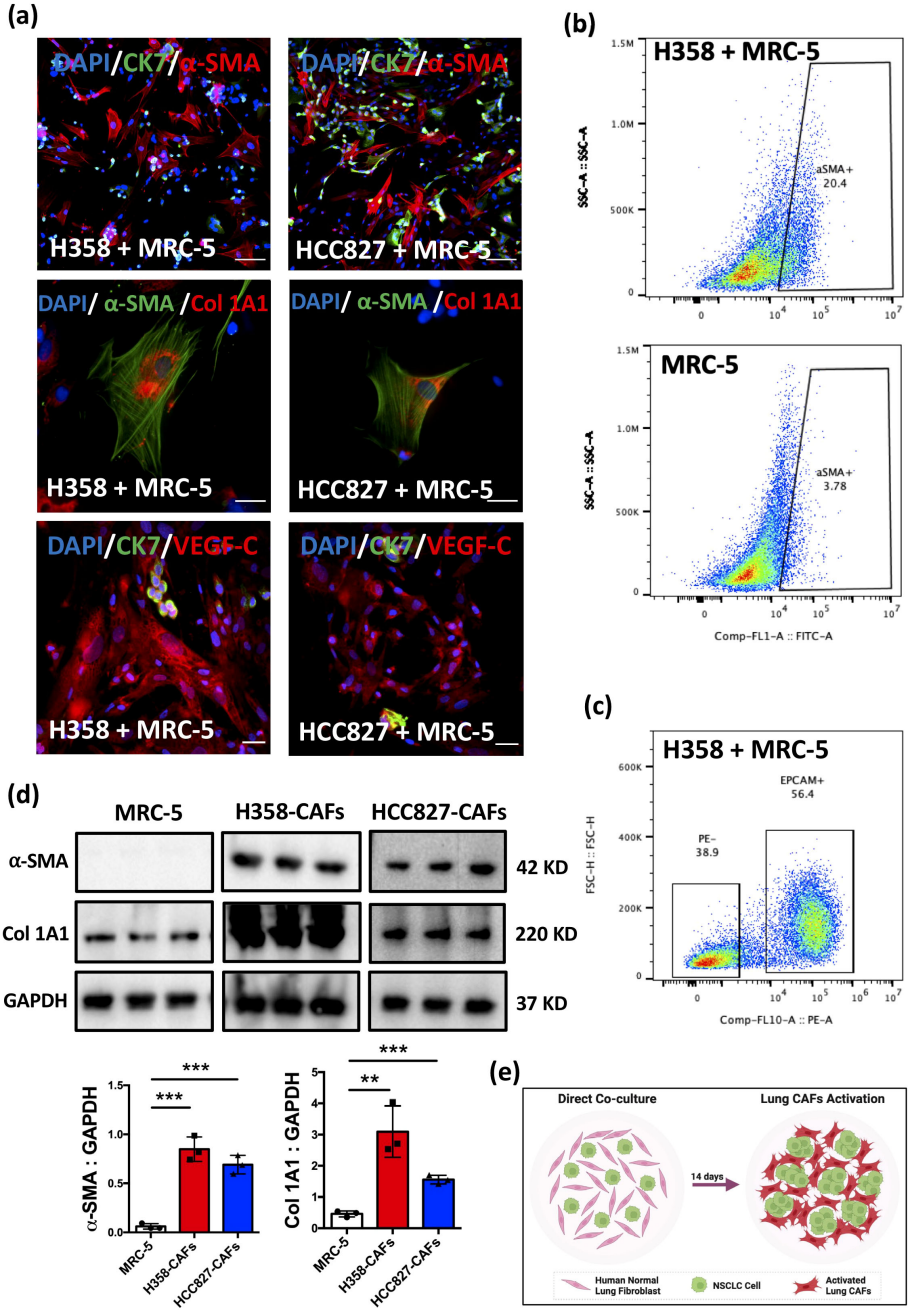
Induction of phenotypic and functional activation in lung CAFs through direct co-culture. **(a)** Representative IF images of staining for α-SMA, CK7, Col 1A1, VEGF-C in MRC-5 cells after co-culture with H358/HCC827 cells for 14 days. DAPI (blue) was used to stain nuclei. Scale bars: 100 μm. **(b)** Representative flow cytometry images showing the percentage of α-SMA+ fibroblasts in MRC-5 and H358 + MRC-5 (co-cultured for 14 days). **(c)** Representative flow cytometry images of cell sorting. EPCAM was used to stain cancer cells. **(d)** α-SMA and Col 1A1 expression in MRC-5, H358-CAFs, and HCC827-CAFs assessed by Western blot. GAPDH served as a loading control. Quantification of α-SMA expression in MRC-5 cells (Mean ± SEM: 0.06 ± 0.01, n=3) compared with H358-CAFs (Mean ± SEM: 0.84 ± 0.07, n=3) and HCC827-CAFs (Mean ± SEM: 0.69 ± 0.05, n=3). Quantification of Col 1A1 expression in MRC-5 cells (Mean ± SEM: 0.46 ± 0.05, n=3) compared with H358-CAFs (Mean ± SEM: 3.09 ± 0.47, n=3) and HCC827-CAFs (Mean ± SEM: 1.55 ± 0.08, n=3). **(e)** Graphic illustration of the direct co-culture system (Figure created in BioRender.com). *P* values were assessed by unpaired, two-tailed Student’s t-test and one-way ANOVA. (**p <0.01, ***p <0.001).

IF analysis revealed elevated expression of α-SMA and collagen type 1A1 (Col 1A1) in MRC-5 cells co-cultured with H358 and HCC827 cells (**Figure 1a**), whereas PDGFRβ (21) and PDPN (8)—markers often associated with CAFs—were not detected (**Supplementary Figure S1e**). Flow cytometry confirmed an increase in α-SMA+ cells in the H358 + MRC-5 co-culture system, with ~20% of fibroblasts acquiring this CAFs marker (21) (**Figure 1b**). Notably, VEGF-C, a key mediator of tumor lymphangiogenesis (35), was markedly upregulated in α-SMA+ CAFs, while VEGF-D (36) remained undetectable (**Figure 1a**, **Supplementary Figure S1e**).

To further characterize these activated fibroblasts, we isolated α-SMA+ CAFs by cell sorting (**Figure 1c**), hereafter designated as H358-CAFs and HCC827-CAFs. Western blot analysis confirmed significant upregulation of α-SMA and Col 1A1 (37–39) in H358-CAFs and HCC827-CAFs compared to parental MRC-5 cells (**Figure 1d**), indicating not only phenotypic transformation but also functional activation, as evidenced by increased collagen production.

Collectively, these findings demonstrate that H358 and HCC827 cells effectively reprogram normal lung fibroblasts into CAFs within 14 days of direct co-culture (**Figure 1e**). The resulting H358-CAFs and HCC827-CAFs exhibit phenotypic and functional properties consistent with primary tumor-derived CAFs (9, 21, 40), supporting their potential use in CAFs-rich models for orthotopic xenograft studies.

### Lung CAFs promote tumor growth, desmoplasia, and lymphangiogenesis *in vivo*

**Figure 2:**
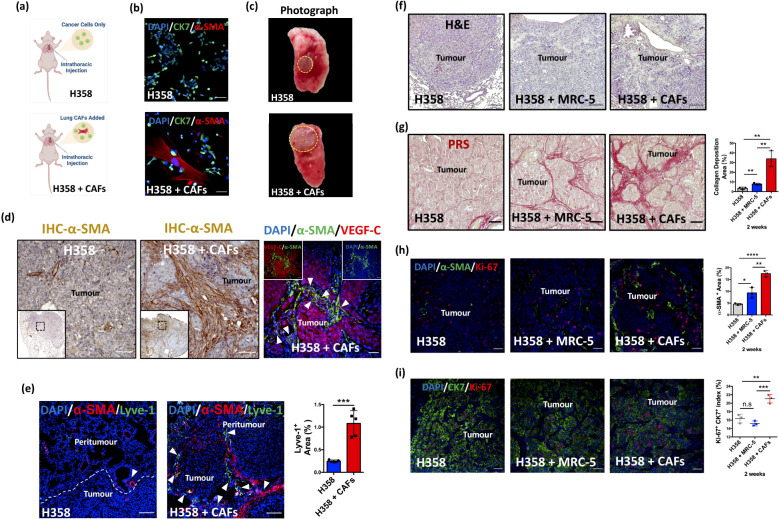
Lung CAFs contribute to desmoplasia and lymphangiogenesis in NSCLC. **(a)** Graphic illustration of intrathoracic injection. (Figure created in BioRender.com). **(b)** Representative IF images of cancer cells (CK7^+^, green) and activated lung CAFs (α-SMA^+^, red). DAPI (blue) was used to stain nuclei. Scale bars: 100 μm. **(c)** Representative photographs of harvested whole left lung with tumour (orthotopic xenografts). **(d)** Representative IHC images of H358/H358 + CAFs orthotopic xenografts stained with α-SMA (brown). Scale bars: 100 μm. Representative IF images of H358-CAFs-added orthotopic xenografts stained with α-SMA (green) and VEGF-C (red) antibodies. DAPI (blue) was used to stain nuclei. Scale bars: 100 μm. **(e)** Representative IF images of orthotopic xenografts stained with α-SMA (red) and lyve-1 (green) antibodies. DAPI (blue) was used to stain nuclei. Scale bars: 100 μm. Quantification of lyve-1 expression area in H358 + CAFs orthotopic xenografts (Mean ± SEM: 1.08 ± 0.13, n=5) compared with H358 only orthotopic xenografts (Mean ± SEM: 0.25 ± 0.01, n=5). **(f)** Representative H&E-stained images of orthotopic xenografts (H358, H358 + MRC-5, and H358 + CAFs), collected 2 weeks post-inoculation. **(g)** Representative PRS (red) images of orthotopic xenografts. Scale bars: 100 μm. Quantification of fibrillar collagen deposition area in H358 + CAFs orthotopic xenografts (Mean ± SEM: 33.88 ± 4.74, n=3) compared with H358 only orthotopic xenografts (Mean ± SEM: 3.420 ± 0.72, n=3). Quantification of collagen deposition area in H358 + CAFs orthotopic xenografts (Mean ± SEM: 33.88 ± 4.74, n=3) compared with H358 + MRC-5 orthotopic xenografts (Mean ± SEM: 7.953 ± 0.53, n=3). Quantification of collagen deposition area in H358 + MRC-5 orthotopic xenografts (Mean ± SEM: 7.953 ± 0.53, n=3) compared with H358 only orthotopic xenografts (Mean ± SEM: 3.420 ± 0.72, n=3). **(h)** Representative IF images of orthotopic xenografts stained with Ki-67 (red) and α-SMA (green) antibodies. DAPI (blue) was used to stain nuclei. Scale bars: 100 μm. Quantification of α-SMA expression area in H358 + CAFs orthotopic xenografts (Mean ± SEM: 17.49 ± 0.77, n=3) compared with H358 only orthotopic xenografts (Mean ± SEM: 4.54 ± 0.23, n=3). Quantification of α-SMA expression area in H358 + CAFs orthotopic xenografts (Mean ± SEM: 17.49 ± 0.77, n=3) compared with H358 + MRC-5 orthotopic xenografts (Mean ± SEM: 9.32 ± 1.29, n=3). Quantification of α-SMA expression area in H358 + MRC-5 orthotopic xenografts (Mean ± SEM: 9.323 ± 1.29, n=3) compared with H358 only orthotopic xenografts (Mean ± SEM: 4.54 ± 0.23, n=3). **(i)** Representative IF images of orthotopic xenografts stained with Ki-67 (red) and CK7 (green) antibodies. DAPI (blue) was used to stain nuclei. Scale bars: 100 μm. Quantification of Ki-67+CK7+ index in H358 + CAFs orthotopic xenografts (Mean ± SEM: 21.07 ± 0.58, n=3) compared with H358 only orthotopic xenografts (Mean ± SEM: 16.34 ± 0.59, n=3). Quantification of Ki-67+CK7+ index in H358 + CAFs orthotopic xenografts (Mean ± SEM: 21.07 ± 0.58, n=3) compared with H358 + MRC-5 orthotopic xenografts (Mean ± SEM: 15.20 ± 0.33, n=3). Quantification of Ki-67+CK7+ index in H358 + MRC-5 orthotopic xenografts (Mean ± SEM: 15.20 ± 0.33, n=3) compared with H358 only orthotopic xenografts (Mean ± SEM: 16.34 ± 0.59, n=3). *P* values were assessed by unpaired, two-tailed Student’s t-test and one-way ANOVA. (n.s.: not significant, *p < 0.05, **p < 0.01, ***p < 0.001, ****p < 0.0001).

To evaluate the functional role of H358-CAFs *in vivo*, we performed direct intrathoracic injections of H358 cells, with or without H358-CAFs, into the left lung of nude mice (**Figure 2a**). Prior to inoculation, H358-CAFs activation was confirmed by immunofluorescence (IF) (**Figure 2b**). Tumor formation and progression were monitored longitudinally using micro-CT imaging (**Supplementary Figure S2h**), validating this approach for tracking both initial tumor establishment and subsequent growth dynamics (27).

Histopathological analysis of resected left lung tumors (**Figure 2c**) revealed significantly increased fibrotic stroma in H358 + H358-CAFs tumors compared to H358-only controls (**Supplementary Figure S2i**, **Figure 2f**). Notably, H358-CAFs maintained persistence in the lung microenvironment throughout the experimental period, contrasting with PDX models where CAFs are typically lost (41). Immunohistochemical (IHC) and IF analyses demonstrated substantially higher α-SMA expression in H358 + H358-CAFs tumors (15-20%) versus H358-only (5%) or H358 + MRC-5 (5-10%) groups (**Figures 2d, e, h**), establishing the necessity of incorporating activated CAFs in orthotopic xenograft (OX) models (13). Similar α-SMA enhancement was observed in HCC827-CAFs co-injection experiments (**Supplementary Figure S2d**).

CAFs-mediated desmoplasia (20, 42), characterized by excessive fibrillar collagen deposition in the TME (38, 43), underscores their functional significance. Building on our *in vitro* findings of CAFs-mediated collagen production (**Figures 1a, b**), we assessed desmoplastic remodeling *in vivo* using picrosirius red staining (PRS). Tumors co-injected with H358-CAFs or HCC827-CAFs exhibited pronounced fibrillar collagen deposition compared to cancer cell-only controls (**Figure 2g**, **Supplementary Figure S2e**), demonstrating CAFs’ pivotal role in driving extracellular matrix (ECM) remodeling and desmoplasia in the TME.

Proliferation analysis revealed a significantly elevated Ki-67 index in H358 + H358-CAFs tumors (20-22%) versus H358-only controls (15-18%) (**Figure 2i**), indicating CAFs-mediated enhancement of cancer cell proliferation. Intriguingly, tumors with normal fibroblasts (H358 + MRC-5) showed reduced proliferation (14-16%), suggesting tumor-suppressive effects of untransformed fibroblasts (5). These observations underscore the preferential use of activated CAFs but not normal fibroblasts in enhancing the fidelity of the OX model.

Notably, IF analysis demonstrated substantial Lyve-1+ lymphatic vessel expansion in peritumoral regions of H358-CAFs co-injected tumors (**Figure 2e**), with parallel findings in the HCC827-CAFs model (**Supplementary Figure S2d**). Stromal α-SMA+ CAFs in both models showed strong VEGF-C upregulation (**Figure 2d**, **Supplementary Figure S2g**) but no detectable VEGF-D (**Supplementary Figure S2j**), implicating CAFs-derived VEGF-C as a key mediator of tumor-associated lymphangiogenesis. These observations further underscore the involvement of lung CAFs in promoting lymphangiogenesis (44).

Collectively, these results demonstrate that lung CAFs functionally remodel the tumor microenvironment through three synergistic mechanisms: (1) direct enhancement of cancer cell proliferation, (2) desmoplastic ECM remodeling via collagen deposition (45), and (3) VEGF-C-mediated lymphangiogenesis (7, 44). This triad of CAFs-driven effects establishes a pro-metastatic niche that facilitates mediastinal lymph node dissemination in NSCLC.

### Lung CAFs facilitate lymphatic spread of cancer cells to mediastinal lymph nodes

**Figure 3:**
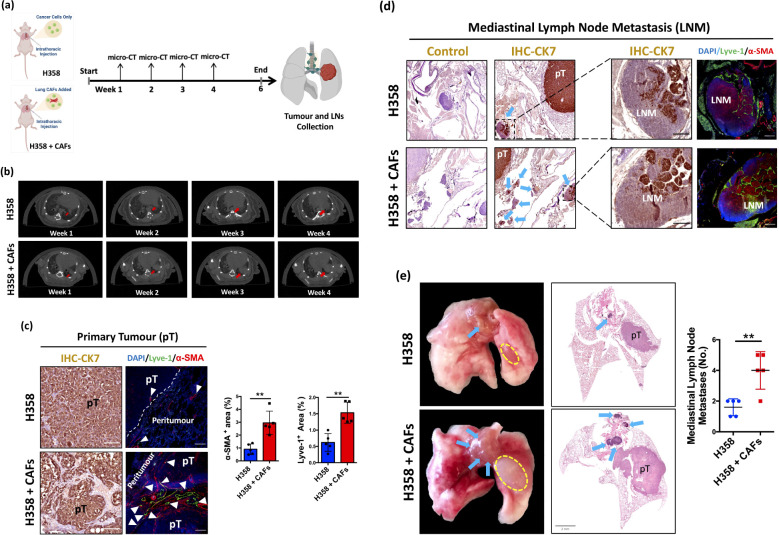
Lung CAFs facilitate mediastinal lymph node metastasis in NSCLC. **(a)** Graphic illustration of intrathoracic injection and flow chart of the experiment. (Figure created in BioRender.com). **(b)** Representative images of weekly micro-CT. Arrows (in red) indicate tumour formation and growth in the left lung of mice. **(c)** Representative IHC/IF images of primary tumour (pT) stained with CK7 (brown), α-SMA (red) and lyve-1 (green) antibodies. Arrows (in white) indicates lymphatic vessels. Hematoxylin (blue) and DAPI (blue) were used to stain nuclei. Scale bars: 100 μm. Quantification of α-SMA^+^ area in H358 + CAFs orthotopic xenografts (Mean ± SEM: 2.95 ± 0.41, n=5) compared with H358 only orthotopic xenografts (Mean ± SEM: 0.91 ± 0.18, n=5). Quantification of lyve-1^+^ area in H358 + CAFs orthotopic xenografts (Mean ± SEM: 1.53 ± 0.14, n=5) compared with H358 only orthotopic xenografts (Mean ± SEM: 0.62 ± 0.12, n=5). **(d)** Representative IHC/IF images of mediastinal lymph node metastasis (LNM) stained with CK7 (brown), α-SMA (red) and lyve-1 (green) antibodies. Hematoxylin (blue) and DAPI (blue) were used to stain nuclei. Scale bars: 100 μm. Arrows (blue) indicates LNM. **(e)** Representative photographs of harvested whole lungs and mediastinum. Representative whole tissue scan images of H&E staining. Dotted line (in yellow) indicates pT. Arrows (in blue) indicates LNM. Quantification of mediastinal lymph node metastases in H358 + CAFs orthotopic xenografts (Mean ± SEM: 4.0 ± 0.56, n=5) compared with H358 only orthotopic xenografts (Mean ± SEM: 1.60 ± 0.25, n=5). *P* values were assessed by unpaired, two-tailed Student’s t-test. (**p <0.01).

To evaluate the role of lung CAFs in facilitating lymphatic metastasis, we extended our analysis to 6 weeks based on previous reports of detectable lymph node metastases in H358 orthotopic xenografts at 4 weeks (16). Serial micro-CT imaging confirmed progressive tumor growth throughout the study period (**Figures 3a, b**).

Histopathological analysis revealed significantly increased α-SMA+ CAFs infiltration and Lyve-1+ lymphatic vessel density in primary tumors (pT) from H358-CAFs co-injected xenografts compared to H358-only controls (**Figure 3c**), confirming the establishment of robust CAFs-rich, lymphangiogenic tumor models. Most notably, both H358-CAFs and HCC827-CAFs co-injection groups demonstrated substantially higher incidence and burden of mediastinal lymph node metastases compared to respective cancer cell-only xenografts (**Figures 3d, e**, **Supplementary Figure S2f**).

### ABT-199 selectively targets lung CAFs *in vitro*

Enhanced apoptotic sensitivity has been extensively documented in various activated cell states, such as α-SMA^+^ fibroblasts (46, 47). Upon differentiation from normal fibroblasts, activated α-SMA^+^ fibroblasts exhibit heightened mitochondrial priming, rendering them more susceptible to apoptosis and relying more on anti-apoptotic proteins for survival (46, 48). This heightened apoptotic sensitivity is closely linked to their enhanced ability to initiate the mitochondrial pathway of apoptosis, primarily governed by the Bcl-2 family of proteins (49, 50). In line with these findings, ABT-199, a Bcl-2 inhibitor known for its pro-apoptotic effects, has been developed and demonstrated efficacy in eliminating α-SMA^+^ fibroblasts (34, 51). Nevertheless, its effectiveness in depleting lung CAFs *in vitro* remains to be fully elucidated.

**Figure 4:**
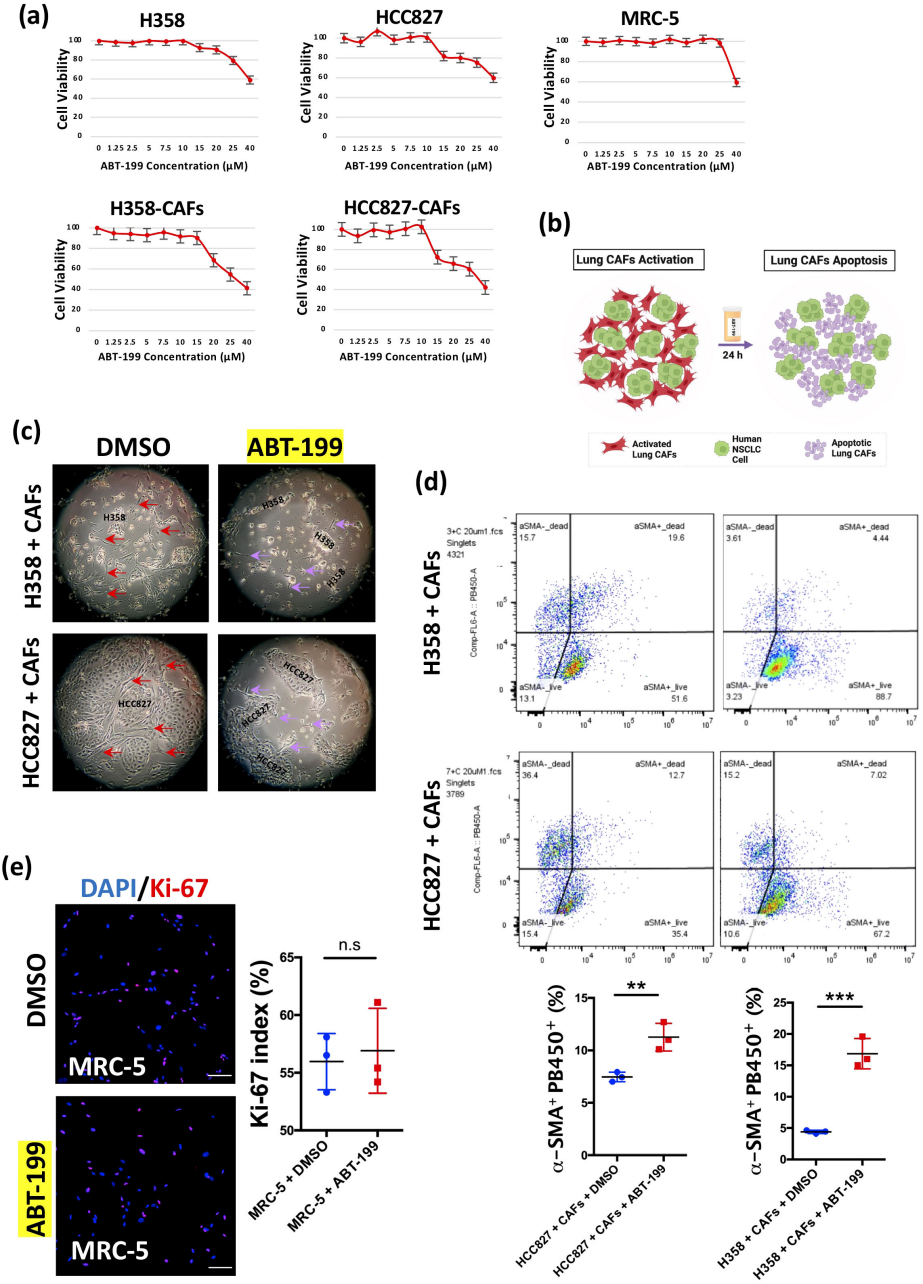
Elimination of lung CAFs by ABT-199 *in vitro*. **(a)** Cell viability (MTT assay, biological triplicates) of cell lines (H358, HCC827, MRC-5) and H358-CAFs and HCC827-CAFs treated with ABT-199 (0-40 μM) for 24 hours. **(b)** Graphic illustration of apoptosis in activated lung CAFs by ABT-199 (Figure created in BioRender.com). **(c)** Representative light microscopic images of H358 + CAFs and HCC827 + CAFs after treatment with DMSO or ABT-199 (20 μM) for 24 hours. **(d)** Representative flow cytometry images showing the percentage of α-SMA^+^PB450^+^ fibroblasts (apoptotic lung CAFs) after treatment with ABT-199 (20 μM) for 24 hours in H358 + CAFs and HCC827 + CAFs compared to treatment with DMSO. Quantification of the percentage of α-SMA^+^PB450^+^ fibroblasts after treatment with ABT-199 (20 μM) (Mean ± SEM: 16.87 ± 1.40, n=3) for 24 hours in H358 + CAFs compared to treatment with DMSO (Mean ± SEM: 4.40 ± 0.15, n=3); Quantification of the percentage of α-SMA^+^PB450^+^ fibroblasts after treatment with ABT-199 (20 μM) (Mean ± SEM: 11.27 ± 0.76, n=3) for 24 hours in HCC827 + CAFs compared to treatment with DMSO (Mean ± SEM: 7.46 ± 0.26, n=3). **(e)** Representative immunofluorescence images of MRC-5 cells stained with Ki-67 (red) antibody after treatment with ABT-199 (20 μM) (Mean ± SEM: 55.97 ± 1.411, n=3) compared to treatment with DMSO (Mean ± SEM: 56.90 ± 2.128, n=3) for 24 hours. DAPI (blue) was used to stain nuclei. Scale bars: 100 μm. *P* values were assessed by unpaired, two-tailed Student’s t-test. (n.s., not significant, **p < 0.01, ***p < 0.001).

Our MTT assays revealed a striking differential sensitivity: while H358, HCC827, and MRC-5 cells showed minimal response, both H358-CAFs and HCC827-CAFs demonstrated dose-dependent apoptosis (**Figures 4a, b**). This selectivity was confirmed in co-culture systems, where 24-hour ABT-199 treatment eliminated most α-SMA+ CAFs while sparing cancer cells (**Figure 4c**). Flow cytometry quantified this effect, showing significant depletion of α-SMA+ populations (**Figure 4d**). Importantly, normal MRC-5 fibroblasts maintained proliferation rates despite treatment (**Figure 4e**), confirming the therapeutic window between activated CAFs and untransformed cells.

### ABT-199 suppresses tumor growth and lymphangiogenesis *in vivo*

**Figure 5:**
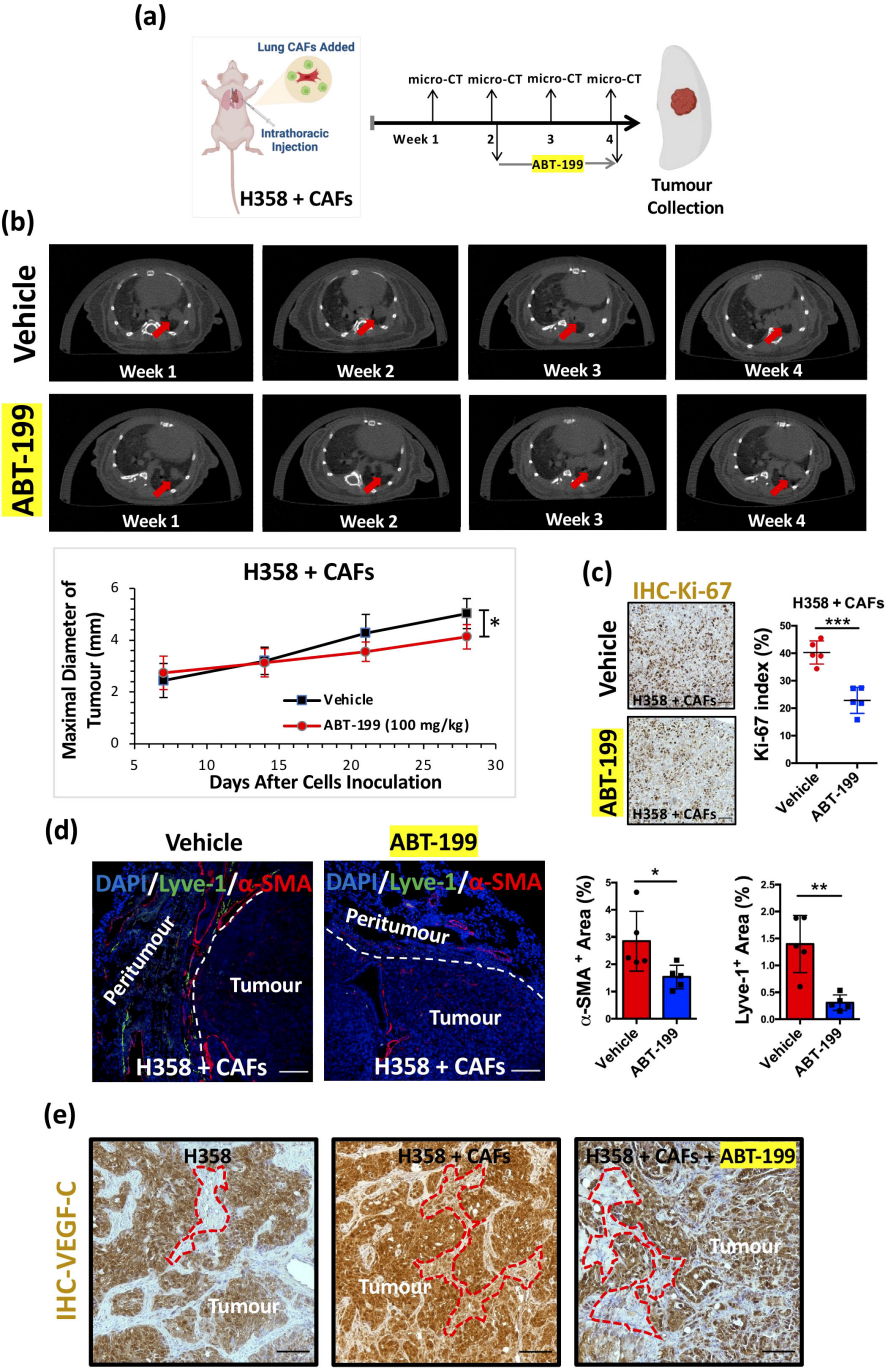
Suppression of tumour growth and lymphangiogenesis by ABT-199 *in vivo*. **(a)** Graphic illustration of intrathoracic injection and experimental flowchart (Figure created in BioRender.com). **(b)** Representative images of weekly micro-CT scans. Red arrows indicate tumour formation and growth in the left lung of mice. Quantification of the maximal diameter of H358 + CAFs orthotopic xenografts treated with ABT-199 (100 mg/kg) (Mean ± SEM: 4.13 ± 0.2, n=5) compared with the vehicle group (Mean ± SEM: 5.03 ± 0.26, n=5). **(c)** Representative IHC images of orthotopic xenografts stained with the Ki-67 antibody. Hematoxylin (blue) was used to stain nuclei. Scale bars: 100 μm. Quantification of the Ki-67 index in H358 + CAFs orthotopic xenografts treated with ABT-199 (100 mg/kg) (Mean ± SEM: 22.86 ± 2.12, n=5) compared with the vehicle group (Mean ± SEM: 40.26 ± 1.90, n=5). **(d)** Representative immunofluorescence images of orthotopic xenografts stained with α-SMA (red) and lyve-1 (green) antibodies. DAPI (blue) was used to stain nuclei. Scale bars: 100 μm. Quantification of the α-SMA+ area in H358 + CAFs orthotopic xenografts treated with ABT-199 (100 mg/kg) (Mean ± SEM: 1.53 ± 0.19, n=5) compared with the vehicle group (Mean ± SEM: 2.85 ± 0.49, n=5). Quantification of the lyve-1+ area in H358 + CAFs orthotopic xenografts treated with ABT-199 (100 mg/kg) (Mean ± SEM: 0.31 ± 0.07, n=5) compared with the vehicle group (Mean ± SEM: 1.40 ± 0.24, n=5). **(e)** Representative IHC images of orthotopic xenografts stained with the VEGF-C antibody. Hematoxylin (blue) was used to stain nuclei. Scale bars: 100 μm. *P* values were assessed by unpaired, two-tailed Student’s t-test (*p < 0.05, **p < 0.01, ***p < 0.001).

To investigate the *in vivo* impact of ABT-199 (100mg/kg) (33), we conducted experiments using the H358 + H358-CAFs OX model (**Figure 5a**). Analysis via micro-CT revealed a significant reduction in tumor size after a 14-day treatment with ABT-199 (**Figure 5b**), indicating its potential to suppress tumor growth. Additionally, IHC analysis demonstrated a marked decrease in Ki-67 index in orthotopic xenografts following ABT-199 administration (**Figure 5c**), suggesting decreased proliferation upon elimination of lung CAFs. Histological examination also illustrated a notable decline in both α-SMA and lyve-1 expression levels in ABT-199-treated H358 + H358-CAFs orthotopic xenografts compared to controls (**Figure 5d**). Additionally, IHC analysis revealed diminished VEGF-C expression in the stromal area of orthotopic xenografts (**Figure 5e**), indicating reduced lymphangiogenic growth factors within the TME following depletion of lung CAFs. These coordinated effects demonstrate that ABT-199-mediated CAFs elimination disrupts the pro-tumorigenic microenvironment, impairing both tumor growth and lymphatic metastasis potential.

### ABT-199 inhibits mediastinal lymph node metastasis through CAFs depletion

**Figure 6:**
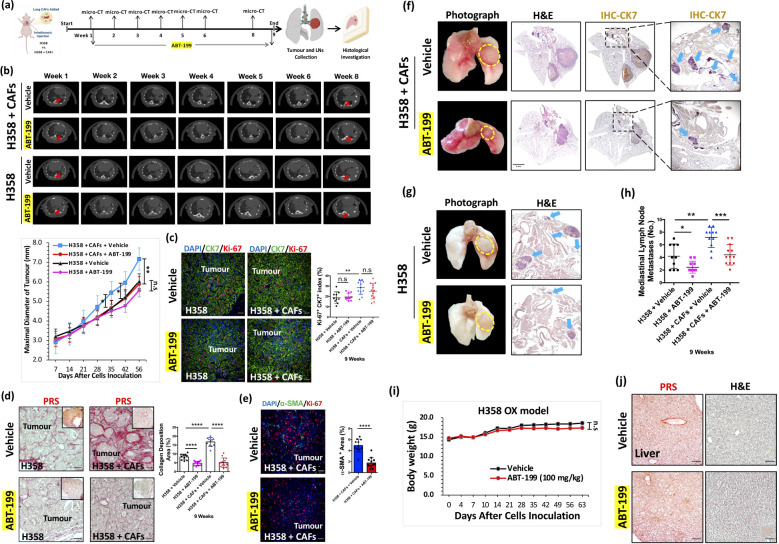
Inhibition of mediastinal lymph node metastasis by ABT-199 *in vivo*. **(a)** Flow chart of the treatment of ABT-199 in H358 + CAFs and H358-only OX models (Figure created in BioRender.com). **(b)** Representative images of weekly micro-CT. Quantification of maximal diameter of the H358 + CAFs orthotopic xenografts treated with ABT-199 (100mg/kg) group (Week 5: Mean ± SEM: 4.67 ± 0.28, n=12; Week 6: Mean ± SEM: 5.14 ± 0.25 n=12; Week 8: Mean ± SEM: 5.88 ± 0.28, n=12) compared with vehicle group (Week 5: Mean ± SEM: 5.44 ± 0.20, n=11; Week 6: Mean ± SEM: 5.95 ± 0.20,n=11; Week 8: Mean ± SEM: 7.16 ± 0.32, n=11). Quantification of maximal diameter of the H358-only orthotopic xenografts treated with ABT-199 (100mg/kg) group (Week 8: Mean ± SEM: 5.56 ± 0.18 n=11) compared with vehicle group (Week 8: Mean ± SEM: 6.03 ± 0.35 n=10). **(c)** Representative IF images of H358 + CAFs and H358-only orthotopic xenografts stained with Ki-67 (red) and CK7 (green) antibodies. Quantification of Ki-67^+^CK7^+^ index of H358 + CAFs orthotopic xenografts treated with ABT-199 (100mg/kg) group (Mean ± SEM: 23.01 ± 2.14, n=12) compared with vehicle group (Mean ± SEM: 25.21 ± 1.80, n=11). DAPI (blue) was used to stain nuclei. Scale bars: 100 μm. Quantification of Ki-67^+^CK7^+^ index of H358-only orthotopic xenografts treated with ABT-199 (100mg/kg) group (Mean ± SEM: 19.53 ± 1.02 n=11) compared with vehicle group (Mean ± SEM: 18.91 ± 1.71 n=10). DAPI (blue) was used to stain nuclei. Scale bars: 100 μm. **(d)** Representative PRS (red) images of H358 + CAFs and H358-only orthotopic xenografts. Scale bars: 100 μm. Quantification of fibrillar collagen deposition area index of H358 + CAFs orthotopic xenografts treated with ABT-199 (100mg/kg) group (Mean ± SEM: 5.05 ± 0.78, n=12) compared with the vehicle group (Mean ± SEM: 16.75 ± 0.74, n=11). Quantification of fibrillar collagen deposition area index of H358-only orthotopic xenografts treated with ABT-199 (100mg/kg) group (Mean ± SEM: 4.71 ± 0.35, n=11) compared with the vehicle group (Mean ± SEM: 8.33 ± 0.51, n=10). **(e)** Representative IF images stained with Ki-67 (red) and α-SMA (green) antibodies. Quantification of α-SMA^+^ area of H358 + CAFs orthotopic xenografts treated with ABT-199 (100mg/kg) group (Mean ± SEM: 1.76 ± 0.28, n=12) compared with vehicle group (Mean ± SEM: 4.93 ± 0.35, n=11). DAPI (blue) was used to stain nuclei. Scale bars: 100 μm. **(f)** Representative photographs of harvested whole lungs and mediastinum. Scale bars: 2mm. Representative whole tissue scan images of H&E staining and IHC stained with CK7 (brown) antibody. Arrows (in blue) indicates LNM. **(g)** Representative photographs of harvested whole lungs and mediastinum. Representative images of H&E staining. Arrows (in blue) indicates LNM. **(h)** Quantification of mediastinal lymph node metastases in H358 + CAFs orthotopic xenografts treated with ABT-199 (100mg/kg) group (Mean ± SEM: 2.83 ± 0.21, n=12) compared with the vehicle group (Mean ± SEM: 6.82 ± 0.55, n=11). Quantification of mediastinal lymph node metastases in H358-only orthotopic xenografts treated with ABT-199 (100mg/kg) group (Mean ± SEM: 2.45 ± 0.28, n=11) compared with the vehicle group (Mean ± SEM: 4.20 ± 0.61, n=10). **(i)** Quantification of body weight of H358-only mice treated with ABT-199 (100mg/kg) (Mean ± SEM: 17.35 ± 0.60 n=11) for 8 weeks compared with vehicle (Mean ± SEM: 18.59 ± 0.32 n=10). **(j)** Representative PRS (red) and H&E-stained images of liver collected from mice treated with ABT-199 (100mg/kg) for 8 weeks compared with vehicle. Scale bars: 100 μm. *P* values were assessed by unpaired, two-tailed Student’s t-test and two-way ANOVA. (n.s., not significant, *p <0.05, **p <0.01, ***p <0.001, ****p <0.0001).

The observation of only 1–2 mediastinal lymph node metastases in the 6-week H358-only OX model (**Figure 3e**) raised concerns regarding the limited robustness for comparative analysis or definitive conclusions. To address this, we extended the experimental duration to 9 weeks in both the H358 + H358-CAFs and H358-only OX models (**Figure 6a**) to increase the incidence of lymph node metastasis in the control group. Additionally, we initiated ABT-199 treatment at week 1 post-tumor formation and sustained it for 8 weeks (33) in both OX models to assess its potential in inhibiting mediastinal lymph node metastasis (**Figure 6a**).

Longitudinal micro-CT analysis revealed a significant reduction in tumor diameters from weeks 5–8 in the H358 + H358-CAFs group, while no such effect was observed in H358-only xenografts (**Figure 6b**). Importantly, Ki-67 indices remained unchanged in both models (**Figure 6c**), confirming that ABT-199’s antitumor effects were mediated through microenvironmental modulation rather than direct cancer cell cytotoxicity.

PRS analysis demonstrated progressive stromal activation, with fibrillar collagen deposition increasing from <5% at 2 weeks to 5-10% by 9 weeks in H358-only tumors (**Figure 6d**). The H358 + CAFs group showed significantly greater collagen accumulation, highlighting the sustained pro-fibrotic activity of transplanted CAFs. ABT-199 treatment substantially reduced collagen deposition in both models and significantly decreased α-SMA+ areas in H358 + CAFs xenografts (**Figures 6d, e**), demonstrating effective CAFs depletion and consequent disruption of desmoplastic stroma.

Metastatic analysis revealed ABT-199’s potent anti-metastatic activity, with significantly reduced mediastinal lymph node involvement in both models (**Figures 6f-h**). The treatment maintained an excellent safety profile, with no significant body weight changes or evidence of liver toxicity (33) (**Figures 6i, j**).

**Figure 7:**
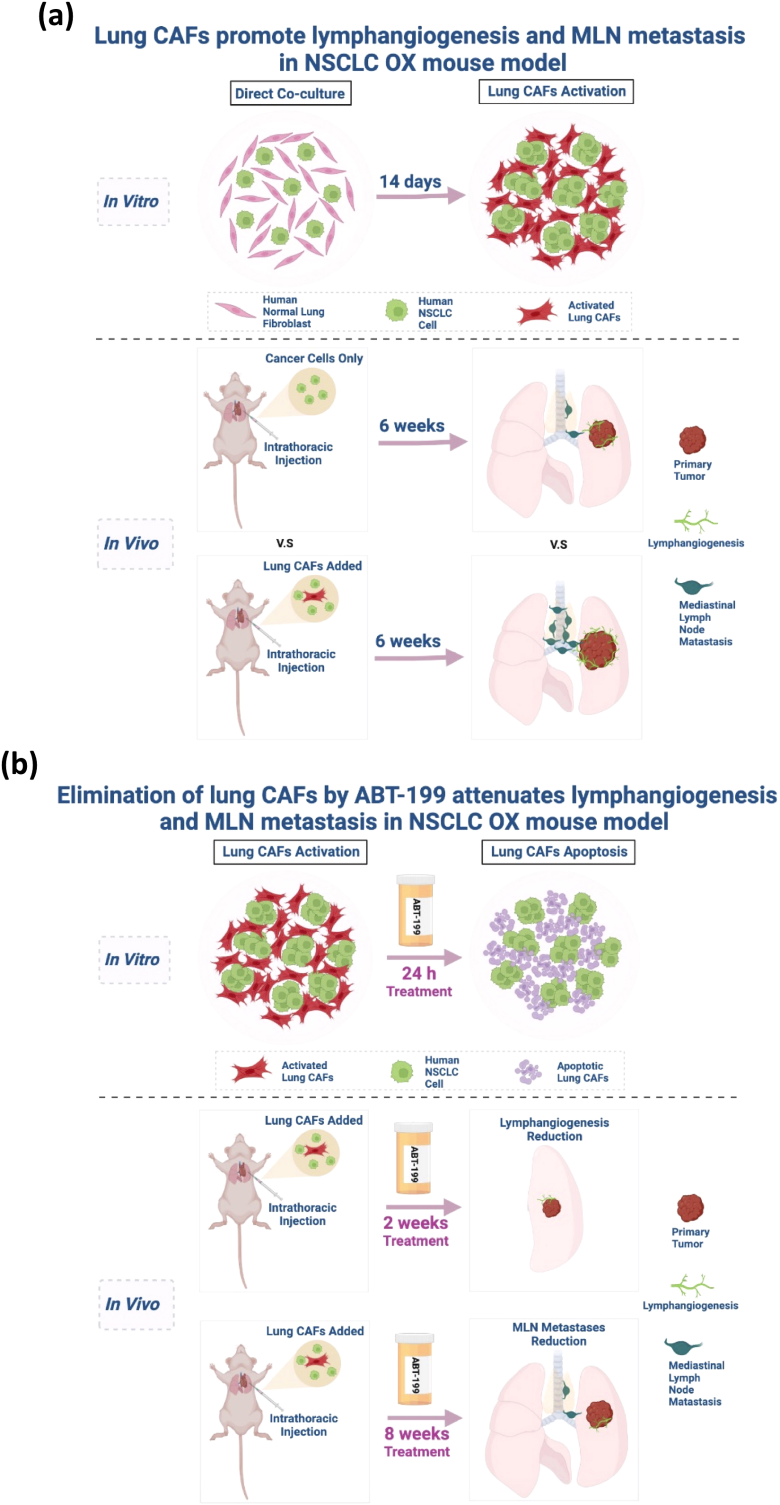
**(a)** Graphic illustration of the activation of lung CAFs *in vitro* and the lymphangiogenic role of lung CAFs in NSCLC *in vivo*. (Figure created in BioRender.com). **(b)** Graphic illustration of the pro-apoptotic effect of ABT-199 on activated lung CAFs *in vitro* and the effectiveness of ABT-199 on NSCLC lymphangiogenesis and mediastinal lymph node metastasis *in vivo*. (Figure created in BioRender.com).

These findings establish that ABT-199 inhibits metastasis primarily through CAFs elimination and subsequent collapse of the pro-metastatic niche, rather than through direct tumor cell effects (**Figure 7**). The compound’s specific activity against CAFs-rich tumors and favorable toxicity profile position it as a promising stroma-targeted therapeutic for preventing NSCLC lymphatic dissemination.

## Discussion

Our study establishes lung CAFs as critical orchestrators of metastatic progression in NSCLC through their multifaceted roles in TME remodeling. The development of a robust CAFs-enhanced orthotopic xenograft model addresses a longstanding limitation in NSCLC research by faithfully recapitulating the desmoplastic stroma (42) and lymphatic metastasis patterns observed in human patients. This model demonstrates particular value for preclinical studies, as it overcomes the weak stromal activation and inconsistent metastasis seen in traditional cancer cell-only xenografts (13). The clinical relevance of our approach is underscored by its recapitulation of the established correlation between α-SMA expression, lymph node metastasis, and poor prognosis in NSCLC patients (10, 52, 53).

Lung CAFs, accounting for the majority of NSCLC TME (8, 9), are different from normal fibroblasts (54). Despite the heterogeneity of these CAFs, α-SMA is still the most universally used biomarker for distinguishing their phenotype from normal fibroblasts when isolating CAFs from tumors (11, 20). Accordingly, our *in vitro* findings revealed that H358-CAFs/HCC827-CAFs, generated from co-culture, exhibited a significant elevation of α-SMA and collagen I expression, providing clear-cut evidence for differentiation between lung CAFs and normal lung fibroblasts. This not only provides evidence that lung CAFs originate from resident lung fibroblasts but also suggests the potential for repeated utilization of these activated cells *in vivo* for functional investigation.

*In vivo*, the subcutaneous xenograft model is commonly employed to investigate the molecular mechanisms of lymphangiogenesis and anti-lymphangiogenic strategies in NSCLC (14). However, the more technically demanding orthotopic xenograft mouse model has been proposed to better resemble the human TME, serving as a superior model for preclinical studies (11, 14, 18). As of now, no clinically available treatment targets lymphangiogenesis, due to a lack of understanding about tumor lymphangiogenesis and a compelling preclinical mediastinal lymph node metastatic mouse model for therapeutic screening (14, 55).

In a clinical context, immunohistochemistry assessed α-SMA expression in lung adenocarcinoma, indicating a significant correlation between increased α-SMA abundance, elevated lymph node metastasis, and reduced 5-year overall survival (OS) rates in patients (52, 53). However, in both traditional cancer cells only (H358/HCC827) orthotopic xenograft mouse model, very little desmoplasia and lymphangiogenesis were generated, suggesting that mouse normal lung fibroblasts might be rarely functionally activated. These findings underscore the necessity of exercising caution when employing the traditional orthotopic xenograft mouse model for drug screening, especially considering the complexities of the TME, notably the influence of CAFs. In comparison, with the presence of lung CAFs in our orthotopic xenograft models, abundant lymphangiogenic and desmoplastic tumors were consistently formed, indicating that these improved models could more closely mimic human lung cancer. This aligns with scRNA-seq analysis, revealing that lung CAFs from solid lung adenocarcinomas express higher levels of collagens compared to ground glass nodules, highlighting the significance of excessive desmoplasia in tumor development (56). Indeed, the high desmoplastic reaction of the primary tumor contributes to cancer cell invasion and distant metastasis (57). Hence, our orthotopic xenograft models, for the first time, provide evidence that lung CAFs directly contribute to desmoplasia through production of collagens (37, 38) to assist lymphangiogenesis. Subsequently, we observed the promotion of mediastinal lymph node metastasis with the assistance of lung CAFs, consistent with findings from investigations on the orthotopic esophageal cancer xenograft mouse model (12). The incorporation of lung CAFs into the orthotopic xenograft model may better replicate the human NSCLC TME, particularly in generating sufficient mediastinal lymph node metastases, thereby enhancing its relevance for pre-clinical investigations. These findings also suggest that targeting lung CAFs could represent a novel strategy for preventing mediastinal lymph node metastasis in NSCLC.

Targeting CAFs as an anti-cancer strategy appears controversial and challenging. For example, metalloproteinase inhibitors (MPIs) failed to demonstrate significant efficacy in clinical trials (58), despite promising preclinical studies revealing the suppressive efficacy of matrix metalloproteinases (MMPs) mainly secreted from CAFs (59). Additionally, SMO inhibitors targeting CAFs blocked the Hedgehog signaling pathway and enhanced chemotherapy efficacy in a mouse model but demonstrated no effects in clinical trials for pancreatic ductal adenocarcinoma (PDAC) (60–62). The contradictory results might be attributed to tissue remodeling during PDAC progression upon Hedgehog inhibition and the dosage of the inhibitors (63, 64). In contrast to pharmacologic strategies, selectively depleting α-SMA^+^ fibroblasts by genetic manipulation in pancreatic cancer models led to more aggressive tumor growth (65). This approach may also impact the cell number and biological function of pericytes and vascular smooth muscle cells expressing α-SMA (66, 67). Notably, relying on a rationale of pharmacological or genetic approaches targeting only one signaling pathway or solely α-SMA^+^CAFs may overlook the heterogeneity and plasticity of CAFs, as recently unveiled by scRNA-seq (24, 68). The lessons learned from the failure of clinical trials targeting CAFs underscore the importance of mouse models that accurately mimic the TME of human cancers (69). Our study demonstrated that targeting pre-apoptotic lung CAFs with ABT-199 resulted in a significant reduction of α-SMA^+^CAFs, fibrillar collagen deposition, lymphangiogenesis, and mediastinal lymph node metastasis. These findings underscore the potential of ABT-199 in targeting CAFs in solid tumors as part of a more nuanced and multifaceted approach to cancer therapy.

While these findings provide substantial mechanistic and therapeutic insights, several important considerations merit further investigation. First, emerging single-cell analyses reveal extensive CAFs heterogeneity (70) that our current model may not fully capture, particularly with regard to immune-modulatory CAFs subsets. Second, the immunocompromised nature of our xenograft system precludes evaluation of potentially critical CAFs-immune cell interactions (71). Finally, the optimal clinical application of this approach may require combination strategies, potentially pairing ABT-199 with either conventional cytotoxic therapies or emerging immunotherapies. Future studies incorporating patient-derived CAFs models and immune-competent systems will help address these questions while further validating the therapeutic potential of this stromal-targeting approach.

This work fundamentally advances our understanding of stromal contributions to NSCLC progression while delivering both a validated preclinical model and a promising therapeutic strategy. By demonstrating that lung CAFs coordinate metastasis through integrated structural, proliferative, and lymphangiogenic mechanisms, we establish stromal reprogramming as a bona fide therapeutic target. The efficacy and specificity of ABT-199 in our models provide strong rationale for its clinical evaluation in CAFs-high NSCLC, particularly as a metastasis-preventive strategy. More broadly, these findings highlight the importance of developing therapies that target not only cancer cells but also their supportive stromal ecosystems.

## Data Availability

The original contributions presented in the study are included in the article/**Supplementary Material**. Further inquiries can be directed to the corresponding author.

## Supplementary material

Supplementary Figure 1 Phenotypic activation of lung CAFs by direct co-culture. **(a)** Representative images of morphologic changes of fibroblasts under light microscope after co-culturing MRC-5 with H358/HCC827/H1993 for 14 days. **(b)** Representative IF images of staining for α-SMA after co-culturing MRC-5 with H358/HCC827/H1993 for 14 days. DAPI (blue) was used to stain nuclei. Scale bars: 100 μm. **(c)** Representative IF images of staining for α-SMA, CK7, Col 1A1, vimentin, VEGF-C and VEGF-D in MRC-5 cells. DAPI (blue) was used to stain nuclei. Scale bars: 100 μm. **(d)** Representative IF images of staining for CK7, VEGF-C and VEGF-D in H358 and HCC827 cells. DAPI (blue) was used to stain nuclei. Scale bars: 100 μm. **(e)** Representative IF images of staining for α-SMA, PDGFRβ, PDPN and VEGF-D after co-culturing MRC-5 cells with H358/HCC827 cells for 14 days. DAPI (blue) was used to stain nuclei. Scale bars: 100 μm.

Supplementary Figure 2 Contributions of HCC827-CAFs to lymphangiogenesis, desmoplasia and mediastinal lymph node metastasis. **(a)** Graphic illustration of intrathoracic injection. (Figure created in BioRender.com). **(b)** Representative IF images of HCC827 cells (CK7^+^, green) and activated lung CAFs (α-SMA^+^, red). DAPI (blue) was used to stain nuclei. Scale bars: 100 μm. **(c)** Representative images of micro-CT. Arrows (red) indicates tumour formation in the left lung of mice. **(d)** Representative images of dissected left whole lung with tumour, H&E staining, and IF staining (CK7, green) of orthotopic tumour. Hematoxylin/DAPI (blue) was used to stain nuclei. Scale bars: 100 μm. Representative IF images of tumour staining by α-SMA (red) and lyve-1 (green) antibodies. DAPI (blue) was used to stain nuclei. Scale bars: 100 μm. Quantification of α-SMA expression area in HCC827 + CAFs orthotopic tumours (Mean ± SEM: 2.52 ± 0.36, n=6) compared with HCC827 only orthotopic tumours (Mean ± SEM: 1.11 ± 0.16, n=6). Quantification of lyve-1expression area in HCC827 + CAFs orthotopic tumours (Mean ± SEM: 2.49 ± 0.52, n=6) compared with HCC827 only orthotopic tumours (Mean ± SEM: 0.35 ± 0.10, n=6). **(e)** Representative PRS (red) images of HCC827 only and HCC827 + CAFs tumour site. Scale bars: 100 μm. Quantification of collagen deposition area in HCC827 + CAFs orthotopic tumours (Mean ± SEM: 4.71 ± 0.40, n=3) compared with HCC827 only orthotopic tumours (Mean ± SEM: 0.93 ± 0.45, n=3). **(f)** Representative images of dissected whole lungs with tumour and mediastinum of HCC827 only and HCC827 + CAFs orthotopic xenografts. Quantification of mediastinal lymph node metastases in HCC827 + CAFs (Mean ± SEM: 5.67 ± 0.33, n=6) compared with HCC827 only (Mean ± SEM: 1.83 ± 0.31, n=6). **(g)** Representative IF images of HCC827 + CAFs orthotopic tumour sites stained by α-SMA (green) and VEGF-C (red) antibodies. DAPI (blue) was used to stain nuclei. Arrows (white) indicate representative VEGF-C^+^α-SMA^+^CAFs. Scale bars: 100 μm. **(h)** Representative images of micro-CT. Arrows (red) indicate tumour formation and growth in the left lung of nude mice. **(i)** Representative images of dissected left whole lung with tumour stained with H&E. Hematoxylin/DAPI (blue) was used to stain nuclei. Scale bars: 100 μm. **(j)** Representative IHC images of HCC827/HCC827 + CAFs orthotopic tumour sites stained by VEGF-D (brown) antibody. Representative IHC images of H358/H358 + CAFs orthotopic tumour sites stained by VEGF-D (brown) antibody. Hematoxylin (blue) was used to stain nuclei. Scale bars: 100 μm. *P* value was assessed by unpaired, two-tailed Student’s t-test. (**p <0.01, ****p <0.0001).
