# Supplementary figures and images for: Lung cancer-associated fibroblasts-mediated collagen deposition drives mediastinal lymph node metastasis in non-small cell lung cancer
Source: Front Oncol. 2025 Jun 12;15:1597585. doi: 10.3389/fonc.2025.1597585 (PMC12198238; doi:10.3389/fonc.2025.1597585)

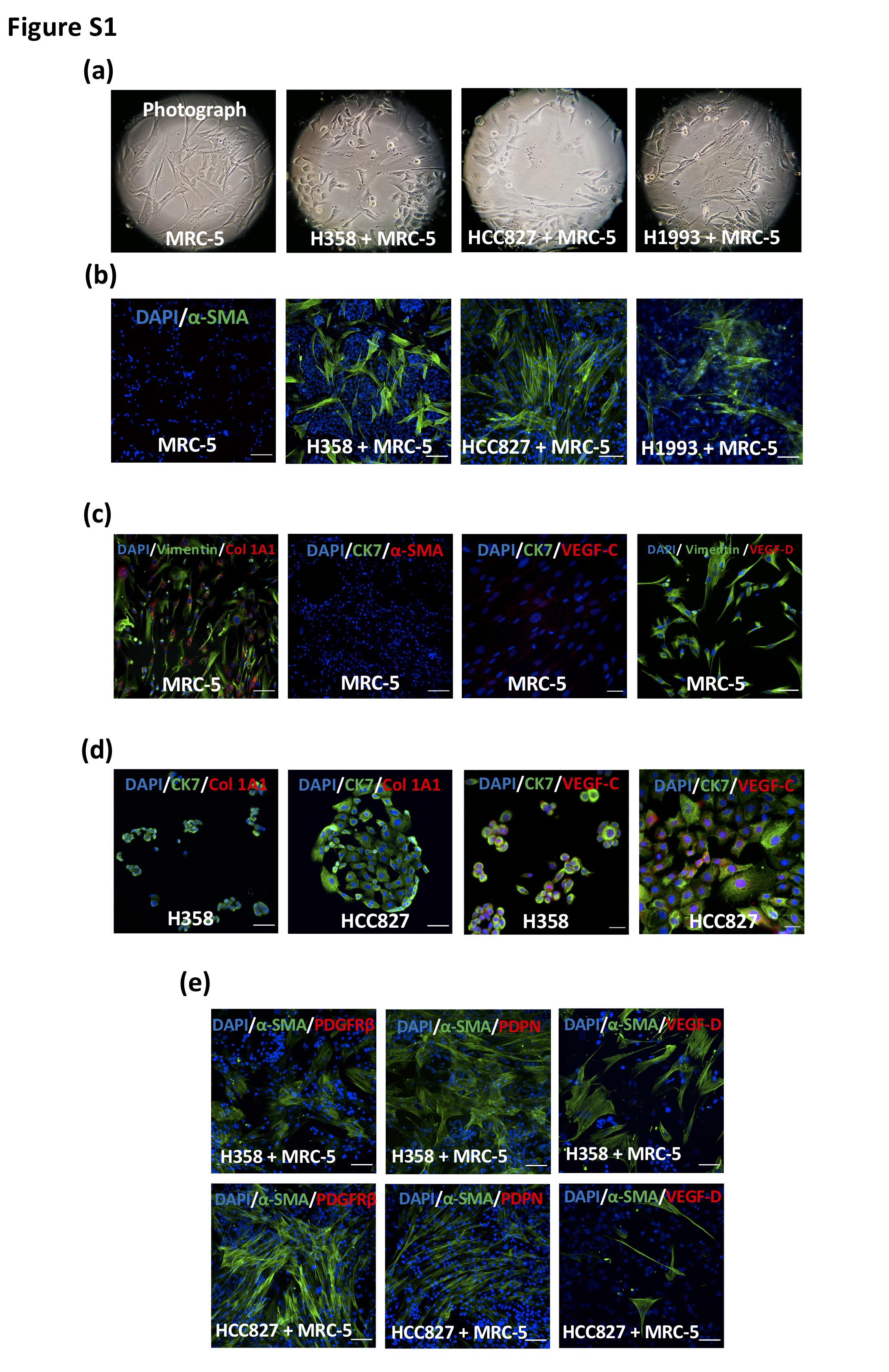

Supplement: Supplementary Figure 1 — Phenotypic activation of lung CAFs by direct co-culture. (a) Representative images of morphologic changes of fibroblasts under light microscope after co-culturing MRC-5 with H358/HCC827/H1993 for 14 days. (b) Representative IF images of staining for α-SMA after co-culturing MRC-5 with H358/HCC827/H1993 for 14 days. DAPI (blue) was used to stain nuclei. Scale bars: 100 μm. (c) Representative IF images of staining for α-SMA, CK7, Col 1A1, vimentin, VEGF-C and VEGF-D in MRC-5 cells. DAPI (blue) was used to stain nuclei. Scale bars: 100 μm. (d) Representative IF images of staining for CK7, VEGF-C and VEGF-D in H358 and HCC827 cells. DAPI (blue) was used to stain nuclei. Scale bars: 100 μm. (e) Representative IF images of staining for α-SMA, PDGFRβ, PDPN and VEGF-D after co-culturing MRC-5 cells with H358/HCC827 cells for 14 days. DAPI (blue) was used to stain nuclei. Scale bars: 100 μm. [file Image1.jpeg]

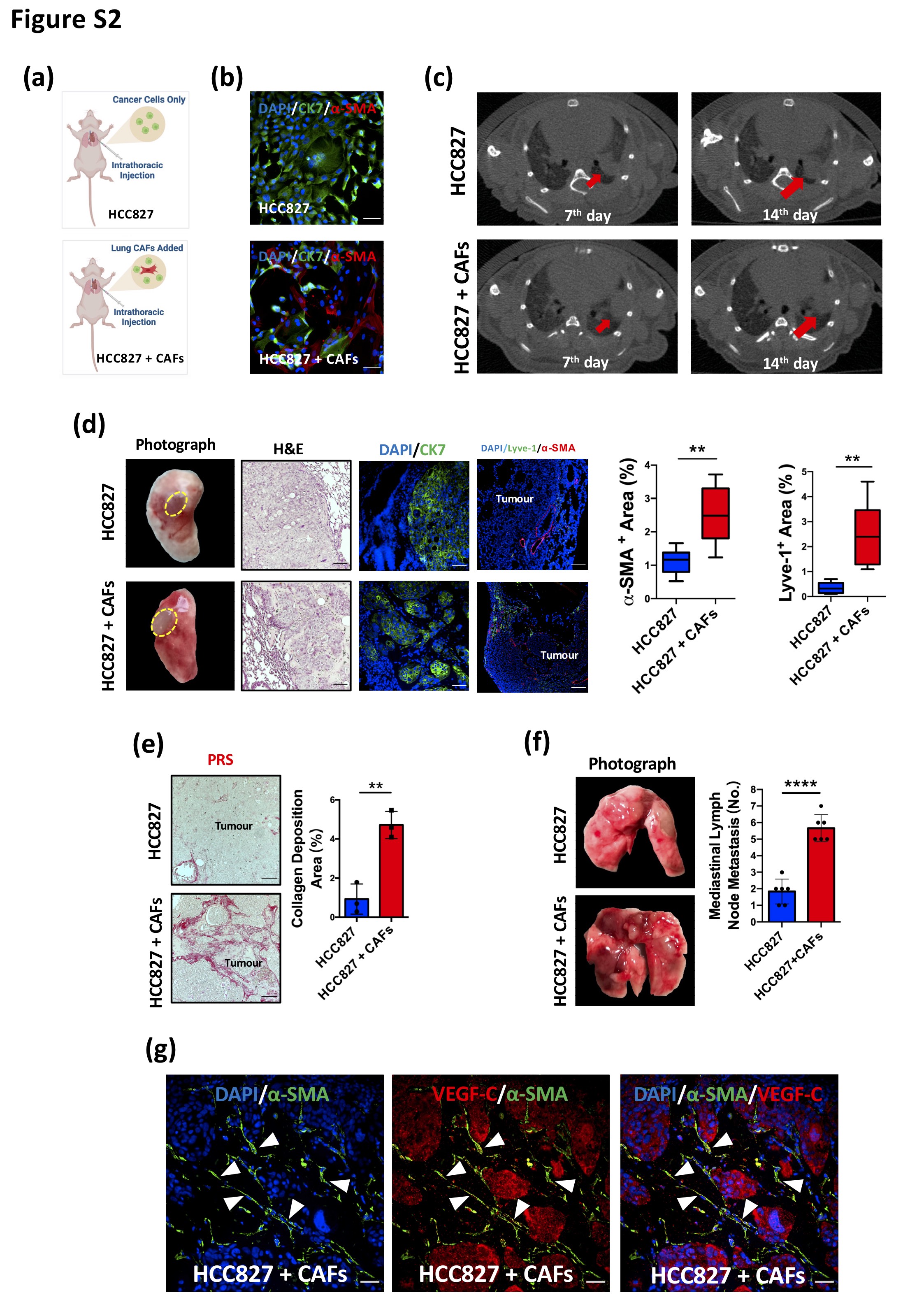

Supplement: Supplementary Figure 2 — Contributions of HCC827-CAFs to lymphangiogenesis, desmoplasia and mediastinal lymph node metastasis. (a) Graphic illustration of intrathoracic injection. (Figure created in BioRender.com). (b) Representative IF images of HCC827 cells (CK7+, green) and activated lung CAFs (α-SMA+, red). DAPI (blue) was used to stain nuclei. Scale bars: 100 μm. (c) Representative images of micro-CT. Arrows (red) indicates tumour formation in the left lung of mice. (d) Representative images of dissected left whole lung with tumour, H&E staining, and IF staining (CK7, green) of orthotopic tumour. Hematoxylin/DAPI (blue) was used to stain nuclei. Scale bars: 100 μm. Representative IF images of tumour staining by α-SMA (red) and lyve-1 (green) antibodies. DAPI (blue) was used to stain nuclei. Scale bars: 100 μm. Quantification of α-SMA expression area in HCC827 + CAFs orthotopic tumours (Mean ± SEM: 2.52 ± 0.36, n=6) compared with HCC827 only orthotopic tumours (Mean ± SEM: 1.11 ± 0.16, n=6). Quantification of lyve-1expression area in HCC827 + CAFs orthotopic tumours (Mean ± SEM: 2.49 ± 0.52, n=6) compared with HCC827 only orthotopic tumours (Mean ± SEM: 0.35 ± 0.10, n=6). (e) Representative PRS (red) images of HCC827 only and HCC827 + CAFs tumour site. Scale bars: 100 μm. Quantification of collagen deposition area in HCC827 + CAFs orthotopic tumours (Mean ± SEM: 4.71 ± 0.40, n=3) compared with HCC827 only orthotopic tumours (Mean ± SEM: 0.93 ± 0.45, n=3). (f) Representative images of dissected whole lungs with tumour and mediastinum of HCC827 only and HCC827 + CAFs orthotopic xenografts. Quantification of mediastinal lymph node metastases in HCC827 + CAFs (Mean ± SEM: 5.67 ± 0.33, n=6) compared with HCC827 only (Mean ± SEM: 1.83 ± 0.31, n=6). (g) Representative IF images of HCC827 + CAFs orthotopic tumour sites stained by α-SMA (green) and VEGF-C (red) antibodies. DAPI (blue) was used to stain nuclei. Arrows (white) indicate representative VEGF-C+α-SMA+CAFs. Scale bars: 100 μm. (h) Repr [file Image2.jpeg]

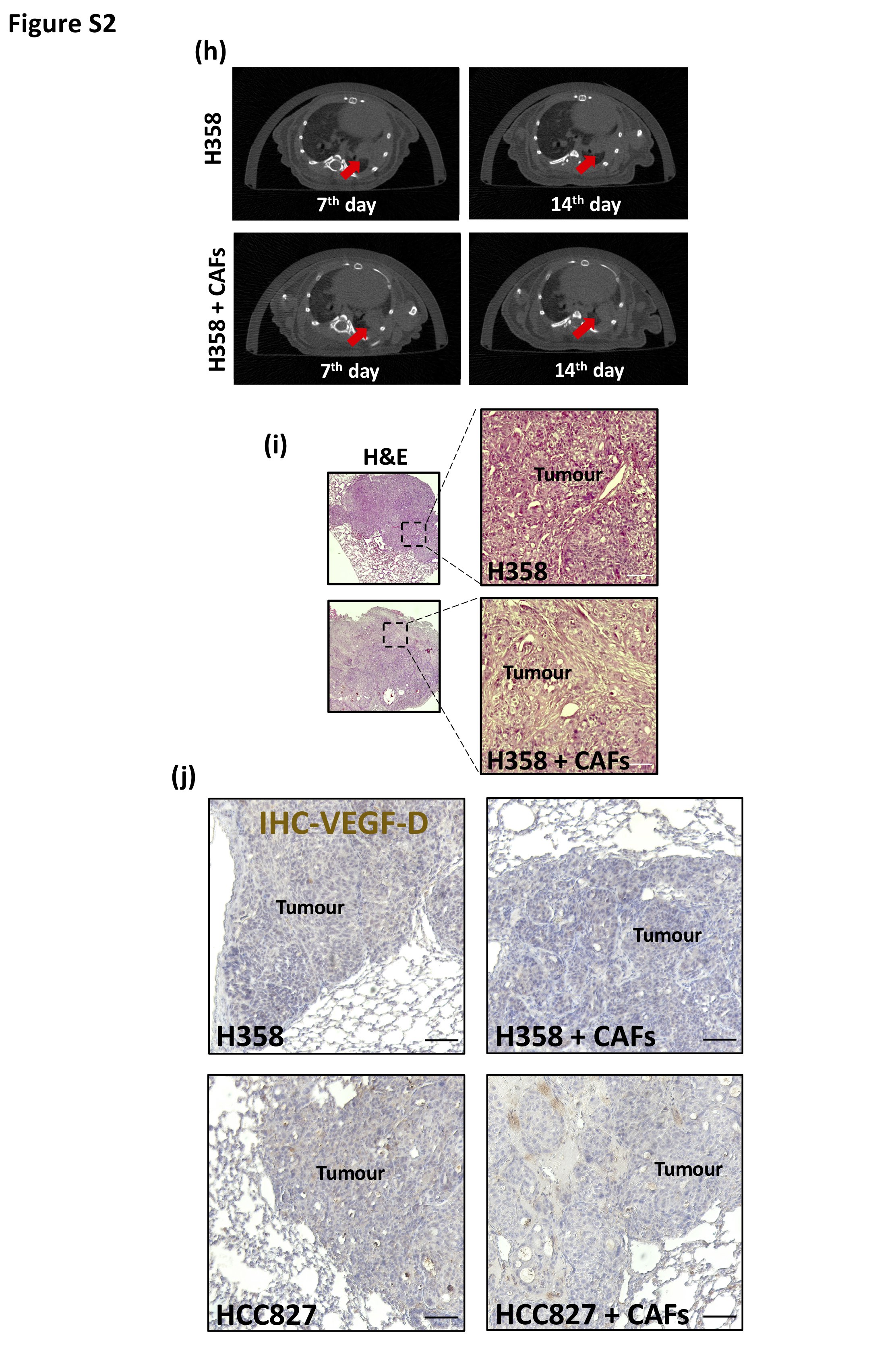

Supplement: Supplementary file 3 [file Image3.jpeg]

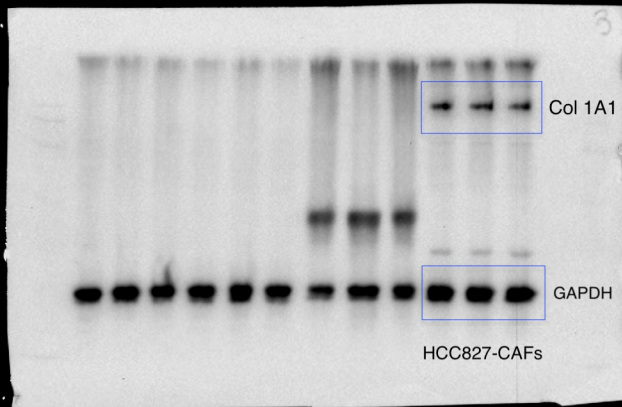

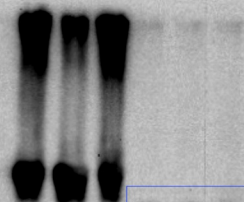

α-SMA

HCC827-CAFs

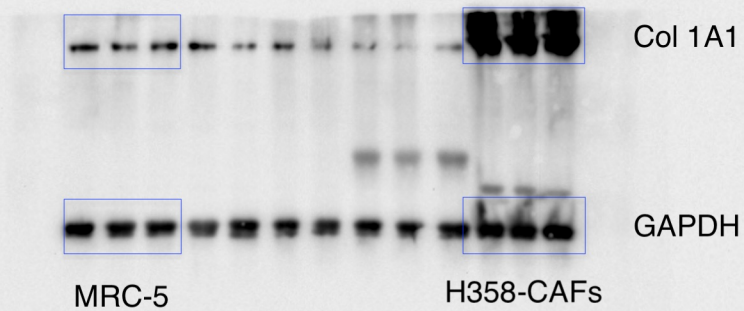

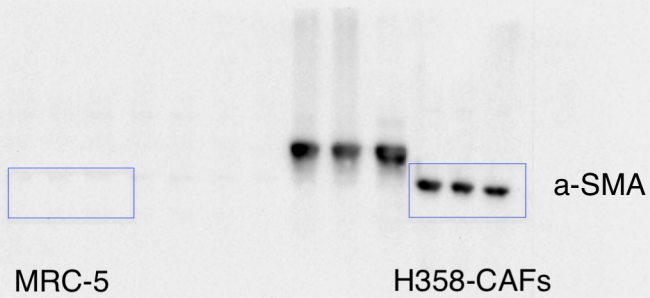

Supplement: Supplementary file 4 [file DataSheet1.pdf]
